# P-1468. Respiratory Syncytial Virus (RSV) prevention product effectiveness against RSV-associated medically attended illness among American Indian and Alaska Native (AI/AN) children in Alaska and the Southwest United States (US), November 2023 – February 2025

**DOI:** 10.1093/ofid/ofaf695.1654

**Published:** 2026-01-11

**Authors:** Laura Hammitt, Joel Espinoza, James Keck, Rachel Hartman, Angela P Campbell, James Chappell, Fatimah S Dawood, Christine Desnoyers, Jennifer Dobson, Natasha B Halasa, Meredith L McMorrow, Maureen Nez, Linda Oxley, Dennie Parker Riley, Mila M Prill, Marqia Sandoval, Rosalyn Singleton, Catherine Sutcliffe

**Affiliations:** Johns Hopkins School of Public Health, Baltimore, Maryland; Johns Hopkins Bloomberg School of Public Health, Baltimore, Maryland; Alaska Native Tribal Health Consortium, Anchorage, Alaska; Johns Hopkins Bloomberg School of Public Health, Baltimore, Maryland; Centers for Disease Control and Prevention, Atlanta, GA; Vanderbilt University Medical Center, Nashville, Tennessee; Centers for Disease Control and Prevention, Atlanta, GA; Yukon-Kuskokwim Health Corporation, Yukon Kuskokwim Delta, Alaska; Alaska Native Tribal Health Consortium, Anchorage, Alaska; Vanderbilt University Medical Center, Nashville, Tennessee; CDC/NCIRD/CORVD/SPB, Atlanta, GA; Johns Hopkins Bloomberg School of Public Health, Baltimore, Maryland; Alaska Native Tribal Health Consortium, Anchorage, Alaska; Johns Hopkins Bloomberg School of Public Health, Baltimore, Maryland; Centers for Disease Control & Prevention, Atlanta, GA; JHSPH, Baltimore, Maryland; Alaska Native Tribal Health Consortium, Anchorage, Alaska; Johns Hopkins, Baltimore, MD

## Abstract

**Background:**

In 2023, nirsevimab, a long-acting monoclonal antibody, was recommended to prevent severe RSV illness in the US for all infants aged < 8 months and children aged 8-19 months at increased risk of severe RSV disease, including AI/AN children. We assessed nirsevimab effectiveness against RSV-associated medically attended acute respiratory infection (MA-ARI) among AI/AN children.

**Figure:**
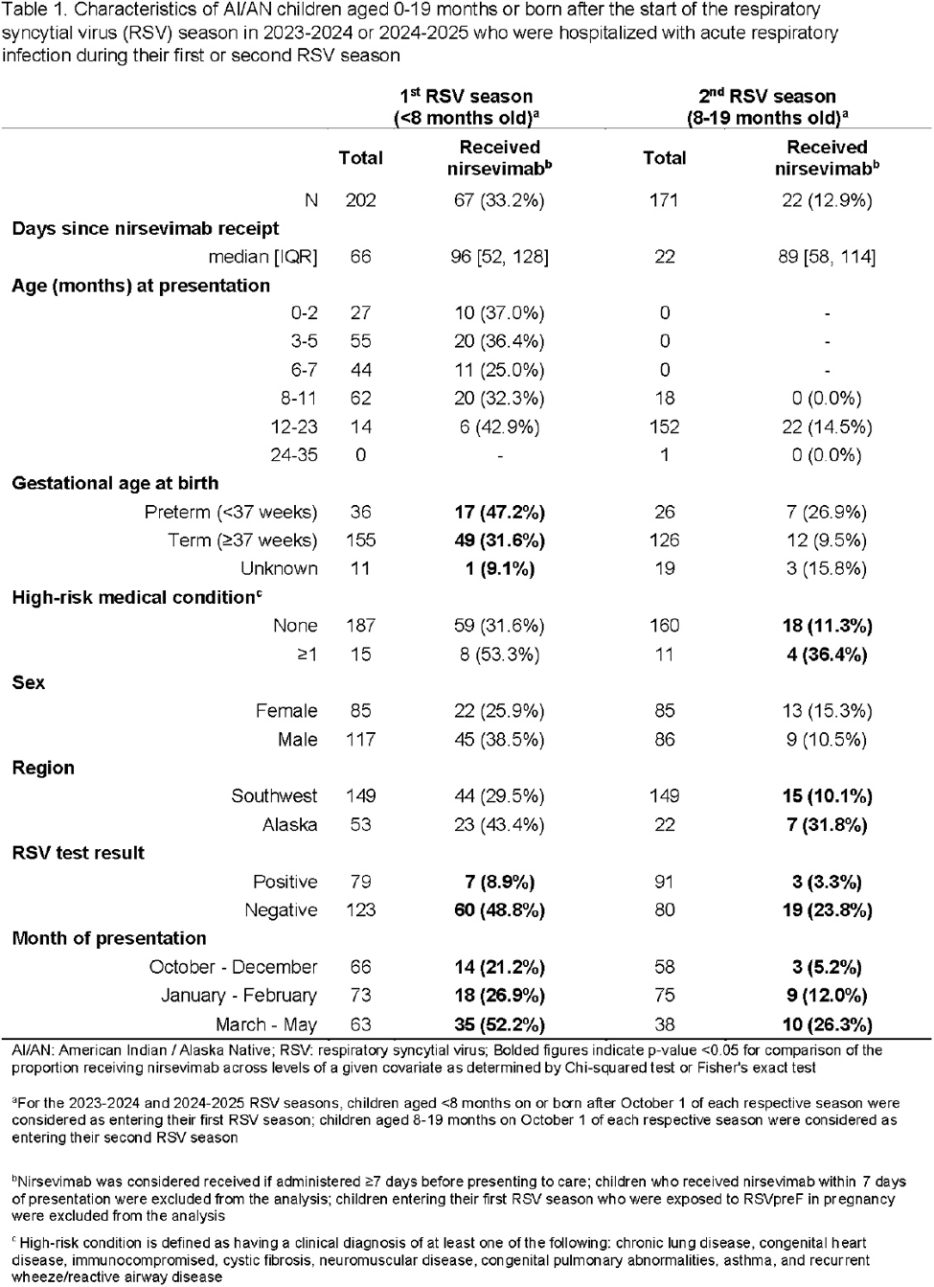

**Figure:**
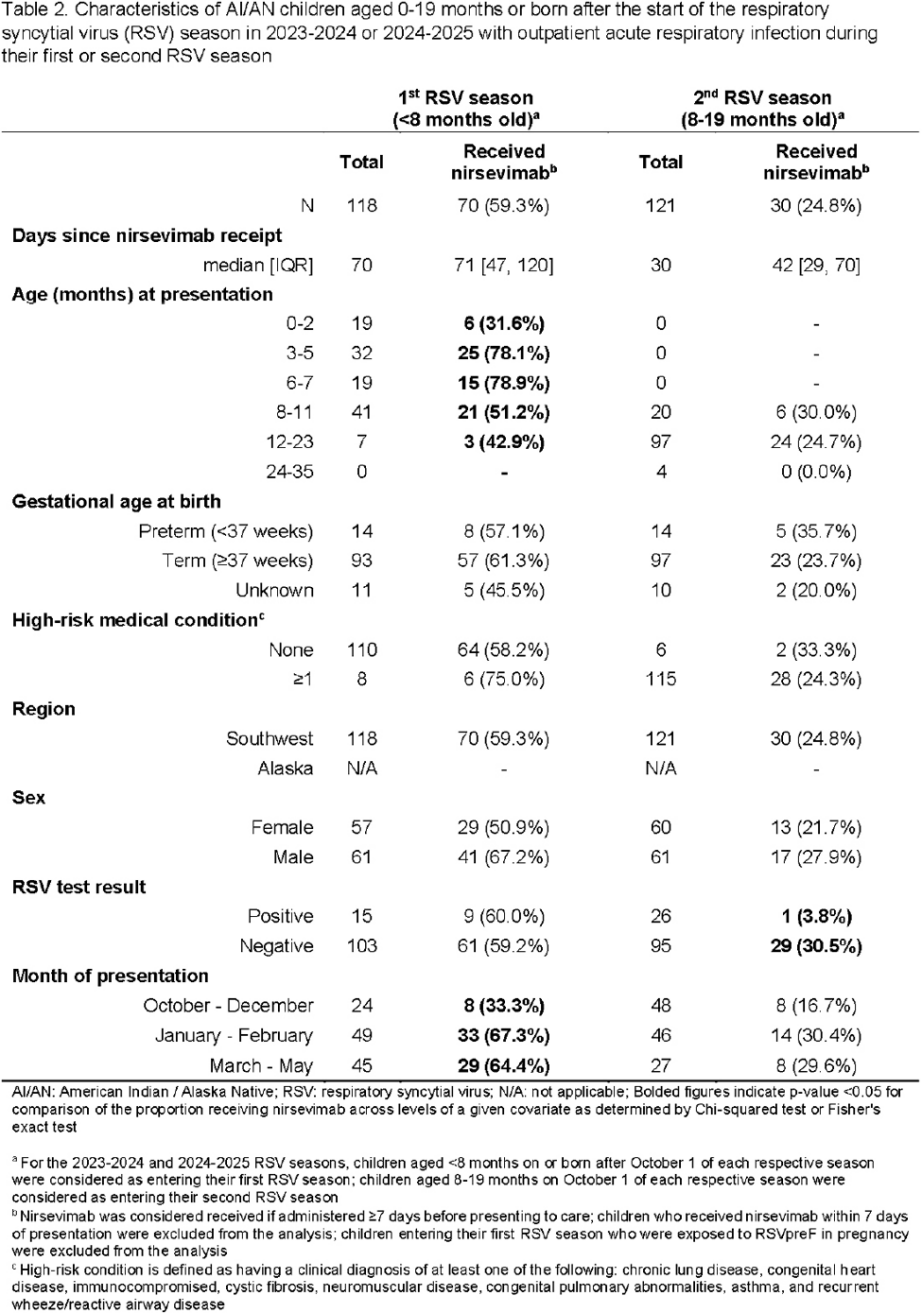

**Methods:**

AI/AN children with MA-ARI were enrolled in the Southwest US and Alaska during Nov 6, 2023-May 31, 2024 and Oct 1, 2024-Feb 28, 2025. Mid-turbinate nasal swabs were tested by polymerase chain reaction for RSV. Exposure to RSV prevention products was confirmed by medical record review. Children exposed to nirsevimab within 7 days of MA-ARI or maternal RSV vaccine were excluded. Nirsevimab effectiveness against hospitalization and outpatient visits was assessed separately for children in their 1^st^ (aged < 8 months on Oct 1^st^ or born during the RSV season) and 2^nd^ (aged 8-19 months on Oct 1^st^) RSV seasons. Firth’s logistic regression estimated adjusted odds ratios (aOR) comparing receipt of nirsevimab by case (RSV-positive) and control (RSV-negative) status adjusting for presence of at least one high-risk condition, sex, enrollment month, and region (Southwest or Alaska). Effectiveness was calculated as (1-aOR)*100%.

**Figure:**
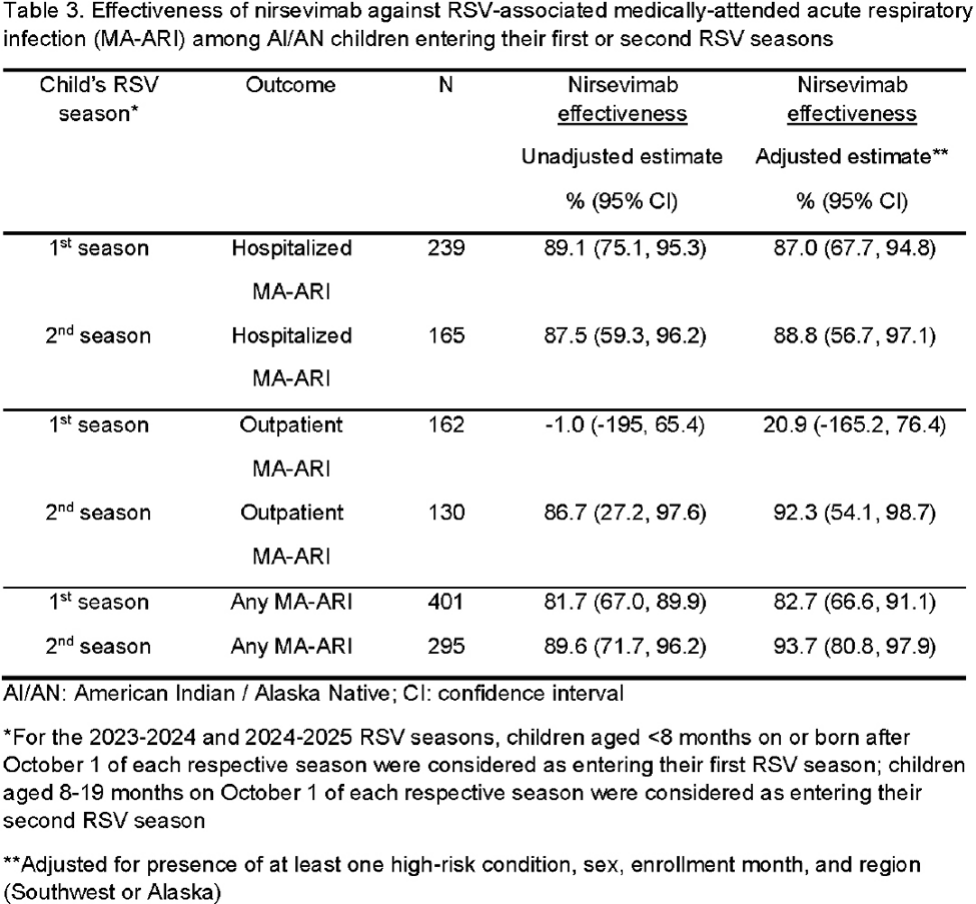

**Results:**

Of 612 children (61% inpatient) with MA-ARI, 94 (29%) in their 1^st^ RSV season and 117 (40%) in their 2^nd^ season had a positive RSV test; 137 (43%) in their 1^st^ and 52 (18%) in their 2^nd^ season received nirsevimab at a median of 80 [IQR: 47, 123] and 59 [IQR: 34, 106] days before illness onset, respectively (Tables 1, 2). Nirsevimab was effective against overall RSV MA-ARI (82.7% [95% CI: 66.6, 91.1] in the 1^st^ season; 93.7% [80.8, 97.9] in the 2^nd^ season) and hospitalization (87.0% [67.7, 94.8] 1^st^ season; 88.8% [56.7, 97.1] 2^nd^ season) (Table 3). Nirsevimab was effective against outpatient RSV MA-ARI among children in their 2^nd^ season (92.3% [54.1, 98.7]) but not their 1^st^ (20.9% [-165.2, 76.4]).

**Conclusion:**

Nirsevimab prevented RSV-associated hospitalization among children in their first and second RSV seasons and RSV-associated outpatient visits among children in their second RSV season, supporting current CDC recommendations for nirsevimab use.

**Disclosures:**

Laura Hammitt, MD, AstraZeneca: Grant/Research Support James Chappell, MD, PhD, Merck: Grant support for etiologic studies of acute respiratory illness in hospitalized children, Amman, Jordan Natasha B. Halasa, MD, CSL-Seqirus: Advisor/Consultant|Merck: Grant/Research Support Catherine Sutcliffe, PhD, SCM, AstraZeneca: Grant/Research Support

